# To Bring up Children

**Published:** 1858-01

**Authors:** 


### TO BRING UP CHILDREN.

UNTIL it has completed its third year, the child should be kept in doors, in high, airy, dry, warm, sun-lighted apartments, during the whole of the twenty-four hours of every day, summer and winter, except from ten until four, during which time it should be in the open air, every second possible, practicable, and safe.

From entering the fourth year until entering the seventh, children should be compelled to keep out of doors, from soon after breakfast until within half an hour of sundown, and from sundown until after breakfast, they should not be allowed, except on special occasions, to go outside the doors of the family residence.

Until entering the seventh year, no child, boy or girl, should ever be allowed to enter a school-room, for any purposes of secular education, beyond learning the names of letters and figures.

When the seventh year is entered, and until the thirteenth year is entered, the following regulations should be strictly adhered to.

The school-room should be large, high, warm, dry, on the second or third story, with windows on all sides.

The school-hours should be from nine until twelve in the forenoon, and from three until four in the afternoon.

All that is to be learned should be learned during school-hours, and during school-hours only; for while not in the school-room, they should be romping, playing, recreating in the open air, or some sheltered place, from after breakfast until sun-down.

But one branch of study should be learned at a time.

That branch should be well learned before proceeding to another—not only well learned in itself, but in all its more direct bearings.

From entering the thirteenth year, until they cease to go to school, three hours should be spent in the forenoon in study, and two hours in the afternoon, with no studies to be pursued out of the school-room; for more necessary is it than ever that the little remaining time from breakfast until sun-down, taking out five hours study, and one for dinner, should be employed in the open air, or large open rooms, in some form of activity, involving hilarious muscular motion.

From sun-down until after breakfast next morning, the best place for children who have not completed their education, is under the parental roof, not to sew or read a moment beyond eight o'clock, but to be employed in cheerful pastimes, in lively amusements, or in learning some of the numerous domesticities, the knowledge and practice of which will exert such a controlling influence on the happiness of after-life. For that man or woman who is not happy at home, has made a sad failure as to the largest portion of a lifetime.

